# RadioMe: an automated home-based radio, music playlist, and diary reminder system: Report on recruitment, music compilation, and listening, and preliminary testing of heart rate activated music

**DOI:** 10.3389/fpsyg.2025.1627466

**Published:** 2025-10-24

**Authors:** Alexander Street, Paul Fernie, Jörg Christfried Fachner, Patrizia Di Campli San Vito, Nicolas Farina, Ming Hung Hsu, Leonardo Muller, Stephen Brewster, Sube Banerjee, Alexis Kirke, Hari Shaji, Paulo Itaborai, Eduardo Reck Miranda

**Affiliations:** ^1^Anglia Ruskin University, Cambridge, United Kingdom; ^2^University of Glasgow, Glasgow, United Kingdom; ^3^University of Plymouth, Plymouth, United Kingdom; ^4^University of Nottingham, Nottingham, United Kingdom

**Keywords:** dementia, music listening, home-based, agitation, heart rate, automated playlists

## Abstract

**Background:**

One of the leading reasons for early admission to a care home in dementia is the escalation of neuropsychiatric symptoms (NPS), and music listening can help regulate these symptoms.

**Aims:**

RadioMe was a project designed for people living at home with dementia to build a system to help them maintain the highest quality of life there for as long as possible, with three functional components: 1. Streaming radio; 2. Providing pre-recorded spoken diary reminders; 3. interrupting the radio with pre-compiled playlists when a wrist-worn heart rate (HR) monitor detects stress. This article reports on the first two stages of this three-stage project: 1. recruitment, the music compilation process, responses of participants when listening, collection of daily agitation HR and behavioural data, and 2. preliminary testing of HR-activated music.

**Materials and methods:**

In stage 1, a playlist compilation process was co-designed with a lived experience group; HR and behavioural data were collected by participants when agitated to refine the algorithm used for automated music activation; 15 home visits were conducted to compile and test the playlists, collecting video, HR, and autobiographical data in each session to inform on playlist suitability for NPS management. Stage 2 involved installing systems to test automated playlist activation, and informal feedback was gathered on system functionality and user experience.

**Findings:**

The music compilation process enabled the creation of bespoke playlists. Sessional HR and video data had limited utility in supporting the suitability of music for NPS management. The methodology for collecting agitation data from participants failed, and the algorithm was not refined. Researchers compiled playlists with 25 people living with dementia, with a mean age of 73.8 years (*n* = 12 men, 13 women). Ten participants had systems installed to test automated music activation. They found it too complex; system calibration was not sensitive enough, music played at random times, and it became repetitive. The system needs extensive refinement to simplify its operation. The activation of the music needs to be better calibrated. A feasible and effective method of gathering data from participants in their homes is required to refine the algorithm, which must include HR/biodata during milder NPS events, as participants reported these to be more in line with their symptoms.

## Background

Neuropsychiatric symptoms (NPS) are common in people living with dementia and include agitation, anxiety, irritability, depression, aggression, and sleep impairment (Lyketsos et al., 2002). Agitation may be linked to changes in heart rate (HR) and heart rate variability (HRV), with some literature suggesting that there are decreases in HRV during agitation events but concluding that further research is required to reliably establish a correlation, which could amount to identifying signatures of agitation in HR and/or HRV (Deutsch et al., 2021; Liu et al., 2015; Ostergaard, 2025). Approximately one-third of this patient population will have at least one NPS at any given time, and nearly 80% will experience symptoms throughout the course of the disease (Lyketsos et al., 2002). Not all NPS lead to increases in arousal that present behavioural challenges; for example, apathy. However, agitated behaviours are the major contributing factor to early care home placement (Dowson et al., 2019). This suggests that more interventions for managing symptoms, as well as improving mood and quality of life, could be beneficial. The overuse of psychotropic medicines has been discouraged (National Institute for Health and Care Excellence, 2018), and alternative methods have been explored, including music-based interventions (Lineweaver et al., 2022; Livingston et al., 2014).

### Playing the right music at the right time

Some studies have helped illustrate the importance of playing the right music at the right time to manage the symptoms of dementia, as well as improve mood and quality of life. This relies on the music starting or being activated at these moments. Reduced mean pain levels resulted where music was played 30 min prior to peak agitation time (Park, 2010); songs about travel or praising nature helped a man with dementia to transition from home to care home several times per week (Yasuda et al., 2006), and medical technicians played music to residents based on their knowledge of their behaviour and routine and at times when they knew that difficult behaviours occurred (Murphy et al., 2018).

There is some evidence that people living with dementia already use music and associated technology to help manage everyday needs and that bespoke music technology could help induce calm and relaxation when needed (Vidas et al., 2024).

### Technology for music selection and listening

#### Publicly available streaming services with a mood function

Various streaming services offer a ‘mood’ function, providing curated playlists tailored to the listener’s preferences. Additional tools have been developed by streaming services, including Spotify (https://www.moodplayl.ist/mood-playlist-generator-personalised-taste/), Apple Music (https://medium.com/edm-rekords/apple-music-debuts-mood-based-playlists-find-your-mood-18600acfeca8), and Deezer (https://support.deezer.com/hc/en-gb/articles/115004367189-Improve-Your-Deezer-Flow-And-Play-Flow-s-Moods-and-Genres#:~:text=Flow’s%20moods%20is%20our%20way,to%20from%20the%20mood%20wheel), which use music listening history and preferences to develop personalised mood-based playlists.

Spotify offers music taxonomies organised by genre, artist, or mood that subscribers can use for emotional regulation. (Pastukhov, 2022) Studies show that personality traits, emotional states, and user age can also be used to identify listening needs (Carrasco et al., 2020). The authors discuss further situational data that could be considered by music recommender systems, including location, time of day, social context, activity, or weather conditions. This added information may help provide music recommendations relevant to the person with dementia and be used to personalise their online user experience, i.e., by customising the interface according to preferred taxonomies such as mood (Ogino and Uenoyama, 2020).

#### AI and biophysiological data for music selection

Studies have sought to determine the effects of music listening using AI, biometric data such as heart rate, and machine learning. Some examples with healthy controls have reported enhanced task performance (Chiu and Ko, 2017), reduced stress (Liu et al., 2015), and positive influence on mood (Ogino and Uenoyama, 2020). The potential for a machine learning decision tree for music selection that benefits relaxation has been reported (Raglio et al., 2020), which could provide quicker access to the appropriate playlists to achieve the desired state or arousal level. The music selected using the resulting algorithms may have induced comparable relaxation levels to preferred music listening participants (Raglio et al., 2021). Significant positive changes in emotional wellbeing, valence, and sense of meaning were found using music playlists selected and compiled using algorithms that map music onto an arousal-valence spectrum (Sice et al., 2020).

#### Music system functionality and user interface in relation to the severity of symptoms

Some research findings suggest that the primary reason older adults do not use information and communication technology (ICT) is a lack of interest or perceived need (Heart and Kalderon, 2013). Whilst there is an increasing trend in its use, possibly motivated by perceived usefulness, there is a need to support older people in accessing such technologies through simpler interface design, training provision, and demonstrating that it can improve their quality of life (Heart and Kalderon, 2013).

Relevant to user interface design and functionality of music listening systems is the severity of symptoms from dementia, whether cognitive or NPS. Conference proceedings reporting early-stage research discuss the need for simplicity in the interface design of music systems for people living with dementia, but without compromising the functionality. Such design decisions being taken in the context of the severity of symptoms – i.e., mild to moderate ones – would enable the operation of more complex interfaces that offer greater functionality (Wesselink et al., 2022).

Touchscreen tablets offer multimedia and multisensory stimulation, with an intuitive interface operating system design, and have been used to facilitate interactions between people living with dementia and their caregivers through the playing of music. Caregivers reported positive mood changes (*P* = <0.001), demonstrating a potentially valuable tool to help improve quality of life whilst living in their own home (Gilson et al., 2019). Other research reports that touchscreen operation is not possible for some living with dementia, suggesting that devices are made ready for use with minimal operational demands (Peeters et al., 2016).

Reminiscence/Rehabilitation and Interactive Therapeutic Activities (RITA) (Aquilano et al., 2012) is a touchscreen device that supports the care delivered to older people, predominantly those with dementia, in hospital and care settings. It can be used for online communication with loved ones, gaming, viewing photos, and listening to music. Use of RITA has not been widely reported in private homes.

#### Mapping music onto an arousal valence for arousal adjustment

Some researchers suggest that it is now possible for music streaming and mobile application technologies to converge, providing adjunct music-based therapy for affective disorders (Schriewer and Bulaj, 2016). They suggest that music can be mapped onto a plane of varying arousal, which relies on effective MIR by measuring electrodermal activity (EDA) and/or electroencephalogram (EEG) activity in people whilst they listen to different music.

A computer program was developed by researchers to help participants select a suitable musical style that was also personally meaningful (Guétin et al., 2009). A total of 21-min musical sequences were broken down into several phases that drew the listener into a state of relaxation. The researchers refer to this as the ‘U’ sequence method, which works on relaxation by gradually introducing a slower tempo, reduced orchestral arrangement, and decreased volume. They reported significant treatment-related reductions in depression and anxiety in a cohort of nursing home residents with mild to moderate Alzheimer’s disease.

#### Music categorisation based on innate neurological responses

X-System (not commercially available) is a music listening application that predicts the level of excitement or relaxation of music, based on a model of the musical brain and body (Sice et al., 2020). The system processes music and maps it onto an arousal scale, from relaxing to exciting. Music can then be streamed to users in playlists designed to achieve their desired emotional or neuropsychological state. Effects may include modulation and regulation of emotion, mood, bodily movement, and autonomic and endocrine response. The automatic categorisation process is based on the Innate Neurophysiological Response to Music model (INRM) (Malloch and Trevarthen, 2009). The model is primarily concerned with the low- and mid-brain processing of music in areas where responses are largely innate and universal.

Some research suggests that music affects human emotions due to its intrinsic influence on body rhythms such as heart rate and breathing, and that listeners’ feelings resonate with the music they hear (Chan et al., 2009). Researchers aimed to select and deliver music to participants that would influence their blood pressure, heart rate, and respiratory rate, to be listened to for 30 min every night before going to sleep. The music offered to participants was described as lacking syncopation, accented beats, or percussive qualities and was played at a tempo of 60–80 beats per minute. They report statistically significant reductions in all physiological measurements and in depression.

This article reports on the first two stages of this three-stage project: 1. Recruitment and retention, the music compilation process and playlist content, and responses of participants when listening with the researchers in their homes; 2. preliminary testing of the music activation, where systems were installed in the homes of 10 participants to see whether their live HR triggered their playlists at times of stress or agitation. The focus is predominantly on the music compilation and listening component, as this was the component of RadioMe that the Anglia Ruskin University research team undertook and completed.

## Materials and methods

### RadioMe aims and objectives

The objective of RadioMe was to work with people living with dementia and their spouse/family member (participants) living in their own home to build and test an integrated system to help manage neuropsychiatric symptoms and provide daily reminders.

### Overarching aims of the RadioMe system (yet to be tested)

1. To increase quality of life by reducing agitation and prompting performance of vital daily tasks.
2. Alleviate the burden for families caring for people with dementia.
3. Reduce care and clinical interventions.
4. Simplify the operation of an in-home assistive system. We will deliver a prototype aid for people with mild to moderate dementia to provide memory reminders and agitation.

RadioMe aims to map natural voice and music elements onto integrated aids for memory and agitation, creating a unique, practical, and impactful broadcast-style radio as a mode of human-computer interaction to support people with dementia who live at home. The novelty lies in the potential to combine existing broadcast media (radio) with in-home and wearable sensor-based technologies to support the delivery of personalised audio media (playlists).

An example of functionality envisaged for RadioMe, which will require ongoing research and development, is as follows: the user switches on the radio in the morning. It initially sounds like their local station. However, at some point that morning, at the start of a song, a DJ-like voice seamlessly overrides the real DJ and reminds the listener to drink to ensure continued hydration. A little later, the radio reminds the listener to eat lunch. Soon, RadioMe detects that the listener is becoming agitated. It overrides the radio and instead selects a song from the user’s personal playlists, which is known to have a calming effect for them. It continues playing the music until it detects in the HR that the user has calmed down (Note: a manually generated audio file of this example is available here: http://www.alexiskirke.com/wp-content/uploads/2018/03/rm_sim.wav).

### Stage 1 and 2 aims of RadioMe

#### Stage 1

Aim 1: The co-design of the music compilation interview form with a lived experience, Patient Carer and Public Involvement (PCPI) group (See Supplementary Appendix).

Aim 2: Improve the agitation HR algorithm: Install RadioMe systems and a daily agitation chart so that participants could provide researchers with 1. Incidents of agitation or stress they experienced (time of onset, duration, cause, de-escalation); 2. HR recorded events into the system from their HR monitor at the time of agitation or stress, allowing these events to be correlated with the behavioural data.

Aim 3: Playlist compilation using the compilation interview form and procedures identified through PCPI, listening to the music with participants whilst observing HR and behavioural responses, and noting autobiographical associations with the music.

#### Stage 2

Aim 1: Installing RadioMe in participants’ homes and testing the playlist activation using participants’ live HR data. This was achieved using the algorithm that had been refined based on their daily agitation chart and HR data from Stage 1 (Aim 2). The aim was to determine whether the music was activating at times when stress or agitation was occurring.

The article does not report on testing the full RadioMe system and the effects of playlists on NPS – this is intended for Stage 3.

**Figure 1:**
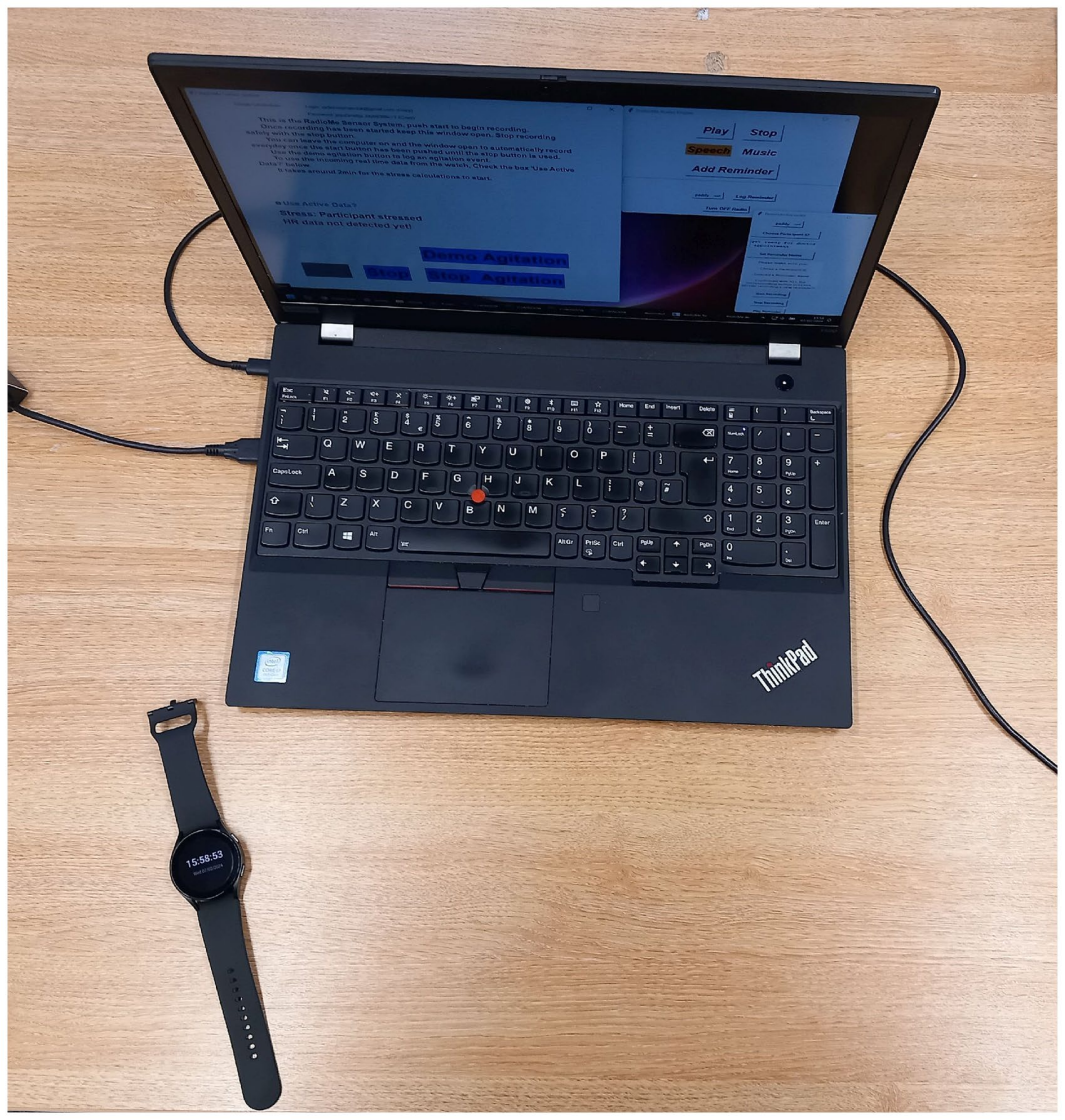
The RadioMe system is housed in a laptop, with the Samsung Galaxy watch also pictured.

The collaborating institutions undertook all technical developments and updates, including building and testing the algorithm and apps that were installed on the smartwatches and laptops (see Figure 1). All articles on the development of the system by partner universities are available, including those by Di Campli San Vito et al. (2023, 2024) and Venkatesh et al. (2021).

### Overview of study design and recruitment aims and objectives

People living with dementia were recruited into this single-arm study for approximately 4 months of weekly music compilation (approximately 15 sessions) and listening sessions in their homes. This was estimated as allowing sufficient time for the researchers to compile and listen to their selected music with them, observe, record, and analyse their behavioural and HR responses. The aim of the video and HR analysis was to monitor consistency of responses, not to answer questions about the cumulative effects of music listening. The objective was to compile playlists of a minimum of 20 tracks, regardless of the duration of each track. The upper limit for the number of participants recruited was 40 people living with dementia (plus their caregiver, if present and willing) over the first two stages of the project.

### Participants

All people contacted had an existing diagnosis of dementia, and no assessments in this regard were conducted by the research team. For screening purposes, each participant underwent the Mini-Mental State Examination (MMSE) in their home, which was delivered by the two researchers conducting home visits to compile and test playlists. Participants were not assessed for agitation, and no data were collected from them about whether they experienced such symptoms – this is intended for Stage 3 of RadioMe.

It was not essential for caregivers to participate in the study, but they were always invited and could potentially assist with daily agitation log data collection (Stage 1, aim 2) and system operation and testing (Stage 2, aim 1). If they agreed to participate, they were provided with a participant information sheet and asked to sign a consent form. No personal data was collected from them, but they could provide informal feedback.

#### Inclusion criteria

1. Diagnosis of dementia (any dementia subtype)
2. Capacity to consent
3. Mini-Mental State Examination score of 10 or more

#### Exclusion criteria

1. Complete hearing loss
2. Diagnosis of Acquired or Congenital Amusia (self-report)

### Recruitment

The majority of participants were recruited from Join Dementia Research (JDR) (https://www.joindementiaresearch.nihr.ac.uk/), which is a UK service connecting people with dementia with researchers, run by the National Institute for Health Research (NIHR) in partnership with Alzheimer’s Research UK. The remaining participants were recruited by NHS partners. The JDR system generated an email notification that was automatically sent to the project manager (Author 1) when someone registered on the system matched the study eligibility criteria and expressed an interest. Some participants were recruited from JDR due to the project manager contacting those registered who were listed as meeting the criteria (as opposed to waiting for an autogenerated e-mail from JDR). Interested parties were visited in their homes and recruited there, once all their questions had been answered and they had decided to participate.

### Stage 1 aim 1

#### PCPI co-design of the playlist compilation form

A PCPI group was formed through the project manager attending a series of face-to-face dementia choir meetings that were held each week in a local church by an external organisation. This group was led by a musician who facilitated the singing of songs from a repertoire requested by people living with dementia who attended, as well as their caregivers. The group was to support social interaction and self-expression. He addressed the group, asking those interested in contributing to the project to come forward. Members came forward at the meetings or contacted the researcher by email, and he coordinated meetings, including agenda items, drafts for review, and payments to the members for their meeting and pre-meeting time, which were in accordance with NIHR policy (NIHR public contributor payment policy|NIHR). Members included five people with lived experience of dementia as professionals and caregivers, some were caring for their spouse or family member at home, whilst others had experienced this and, following care home admission, visited them there.

In a series of online meetings where various drafts of the playlist compilation process were reviewed, the group agreed on a range of prompt questions (Supplementary Appendix) and the following procedural options: 1. Speak to each participant ahead of the first visit to determine music preferences, 2. Where necessary, ask caregivers/spouse in advance about the people living with dementia’s musical preferences, but always encourage the individuals living with dementia to speak for themselves, 3. In the first session, identify any music they have and how they listen to it (CDs, vinyl, smartphone, tablet, Alexa, Radio, and so on), 4. Play music frequently and at the earliest opportunity to help participants recall what is preferred and most personally meaningful 5. Prepare excerpts of songs/music in advance of home visits to prompt further recollection of preferences (one group member had done this for his wife), 6. Use visual prompts if necessary (record/CD covers, pictures of artists that can be displayed on a tablet screen). These procedures were intended to provide the best possible chance for participants to recall music, which is likely to help mitigate stress and agitation at the point of onset, particularly in the face of anticipated memory impairments. The number of sessions offered was also intended to optimise opportunities for preferred music recall.

### Stage 1 aim 2

#### Participant agitation data to improve the music activation algorithm

The second aim in Stage 1 was to improve the algorithm built using HR data from a previously conducted healthy cohort study, which served as the starting point for auto-activation of playlists in RadioMe (Di Campli San Vito et al., 2023). The aim was to invite participants and their caregivers to have a system installed and to record agitation events, for which a daily agitation chart was provided (Supplementary Appendix Figure 2). Whilst wearing the Samsung Galaxy HR Smartwatch, each time the person living with dementia experienced a moment of agitation, they or the caregiver (if observing) were asked to press the ‘agitation start’ button on the laptop and in the daily log write down the date, time, cause, duration, and how it was managed. The details of the event could then be correlated with the HR data and used to help refine the system’s calibration to interrupt radio and begin playing the playlists.

### Stage 1 aim 3

#### Music sessions

A full session description is provided in a separate article (Fernie et al., 2024). Up to 15 sessions were allocated to each participant to ensure adequate time for people to recall their music and autobiographical associations and for researchers to monitor responses. This was to ensure that the compilation process created playlists with the best possible chance of influencing NPS onset during the proposed Stage 3 testing. The article referred to above (Fernie et al., 2024) describes how sometimes relatives/spouses attending sessions with the person living with dementia can inadvertently interrupt the compilation process, and it takes time for the person living with dementia and researchers to identify and remove music that is more the spouse’s preference or wrongly identified by them. The two researchers, visiting participants in their homes to compile and test the playlists, referred to the playlist compilation interview form. They spoke to participants prior to their first session to prepare some initial listening selections, then worked through questions as the sessions progressed.

In sessions, participants were asked if there was a piece of music or an artist they felt like listening to, or if they had a specific question in mind, or to choose from a selection of two to three tracks already identified by them, or from a list displayed on the screen. Alternatively, they played tracks they had already selected, which prompted discussion of that music and led to further tracks being added. This procedure adhered to the recommendations from the PCPI co-design comprising Stage 1 Aim 1. At any point, participants could talk about the music, for example, what it reminded them of or why they liked or disliked it. The researchers tended to ask them about the music, for example, autobiographical associations, before and after listening, rather than during the listening process.

Researchers sometimes sang along to songs and tapped or moved to the music. This was a natural process for each researcher and was not discussed as part of the listening protocol or subjected to analysis. Participants were free to listen to music as they usually would, either alone or socially, between sessions. In some cases, the researchers anticipated that this would be useful and help identify playlist content for the study’s intended purposes.

### HR, video, and autobiographical data for testing the playlists

During the 15 home-based sessions, participants wore a wrist-worn heart rate (HR) monitor (Polar OH-01). The Polar OH-01 wrist-worn device was selected as it collected continuous data, and the app could be downloaded onto a smartphone to enable live monitoring (visible to researchers on their smartphones and on camera in sessions - Supplementary Appendix Figure 1).

The video was recorded using a GoPro camera fitted with an SD card. Video data were transferred directly following each session onto secure university cloud storage, encrypted, and the files were protected. After which, the footage on the SD card was deleted.

HR and video data were later uploaded into the Noldus Observer system, allowing both to be viewed synchronously, which helped determine responses to the music and other events in each session. Given that HR (pulse rate data) is measured within a 1-s range, the manual synchronisation of both time series worked well.

At the start and end of each session, participants underwent a resting state with eyes closed for 3–5 min to observe baseline and post-session HR and to help better identify changes in activity when listening to the music – this was done visually, simply by looking at the HR graph (showing BPM values obtained from pulse rate data) alongside the video data.

### Video data analysis

Videos were tagged from three categories that were agreed upon within the research team based on their review of several sessions with different participants:

Movement: 1. Head, Arm/s, Leg/s, Whole Body, Up Dancing; 2. Audio: Singing, Vocalising, talking about the music (this included related memories or associations), talking (no clear connection with the music, for example, talking about what they needed to do later that day); 3. Emotions: laughing, crying, smiling, and frowning. These were manually tagged in the Observer by the two researchers delivering the sessions. Following the sessions, they would listen through to the songs that had been played, and each time one of the behaviours was observed, they would click on that behaviour, and it would appear on the timeline, as shown in the figures.

HR data from the Polar OH-01 recorded in sessions was not analysed. It was referred to and discussed amongst the team in the context of the literature as a potential indicator of enjoyment of the music, which could be useful where there were no behavioural responses or autobiographical tags.

Comments and feedback from participants on the RadioMe system were not analysed. They are integrated into the results and discussion.

### Stage 2

#### Preliminary testing of HR-activated playlists

Toward the end of playlist compiling and testing, researchers asked participants if they would like to try out the RadioMe system to see how playlists were activated using their live HR. Those who were interested had systems installed for a non-specified period, were shown the setup, and were provided with a QuickStart menu. They were able to contact the researchers to report any errors using the research phone number or by e-mail. Installation took up to 2 h, ensuring that participants went through each step to start the system.

System updates were provided by the research team at the University of Glasgow. These were shared on GitHub (an online platform for creating and sharing files or code facilitating live updates – https://github.com/), enabling the researchers to install and test them on their own RadioMe systems prior to installing them on the participants’ during a home visit.

For this early-stage testing of the RadioMe system, there was no formal data collection method to determine the effects on stress or agitation; only informal, verbal feedback on operation and user experience was available. The system did not time-stamp HR data at points when the music was activated and deactivated, nor log what music was played when the system was activated. These data were intended for collection in Stage 3 testing. If participants were able to get the system running, they would contact the researchers, who would record the problem, solution, and any other relevant comments. If researchers did not hear from participants for a week, they would contact them and ask how the system was running, noting any comments they received.

### RadioMe system and procedures

**Figure 2:**
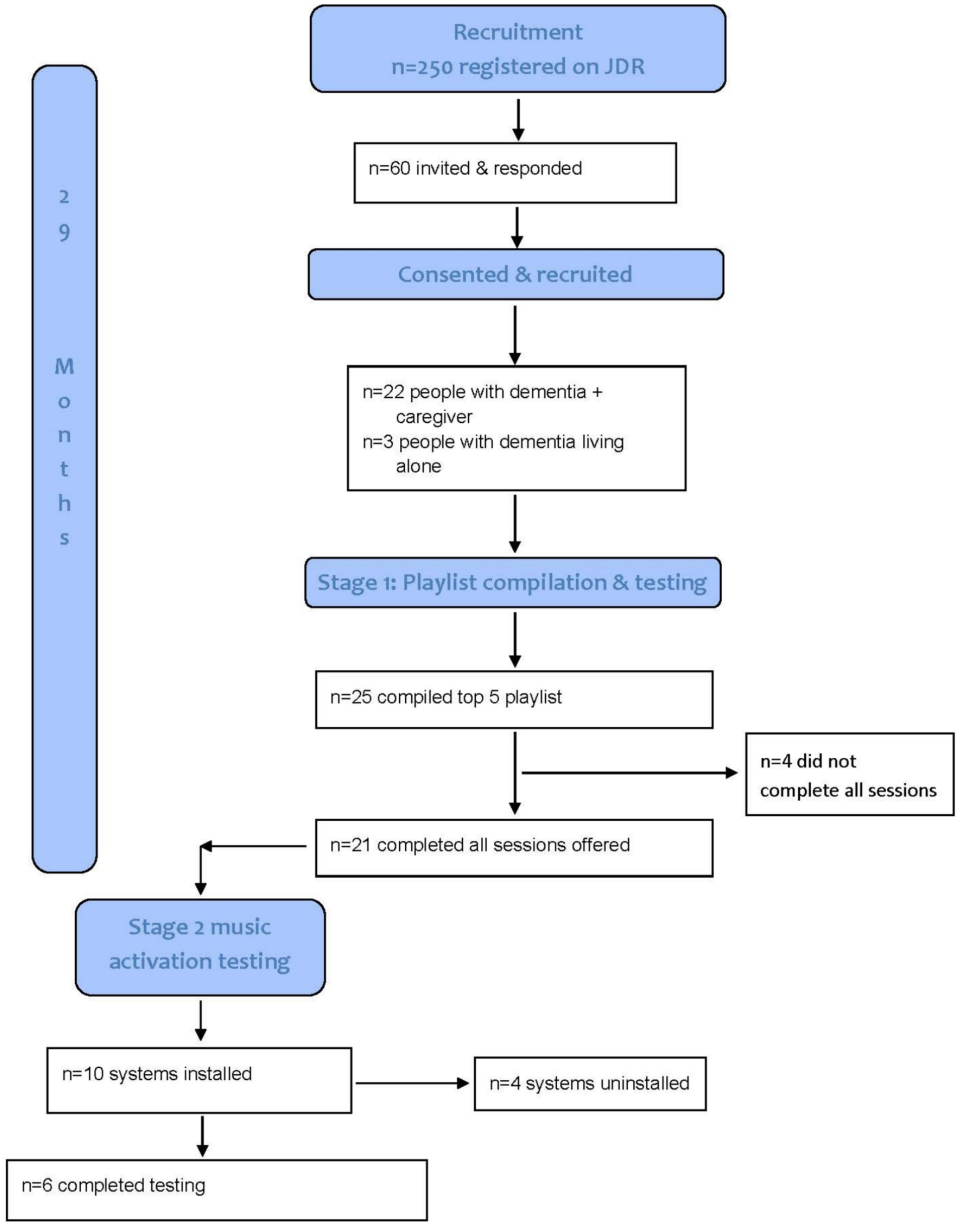
Recruitment and retention consort.

The RadioMe app was housed in a standard Windows laptop (Figure 1), and a full description of it is available in a separate article (Di Campli San Vito et al., 2024). The user clicked on the icon on the desktop, and the program opened. Two windows appeared: the larger one on the left for connecting the watch and the smaller one on the right (at the top) for starting the radio and selecting the playlist. Visible in the larger window on the left was red writing, which disappeared once the laptop received HR data and movement data from the Samsung Galaxy Watch (the third window visible in Figure 2 was added for Stage 3, which is yet to be reported). It contained an audio recorder for creating the spoken diary reminders. The full procedure for starting the system is best followed by referring to the user guide (Supplementary Appendix Figure 3).

Software was developed to fade the radio out when HR indicated stress (Venkatesh et al., 2021), after which a track from the playlist would start. RadioMe also facilitated the pre-recording by researchers of a spoken introduction to the participants’ songs, for example, by fading out the radio. The researcher’s voice would say, for example, ‘Hello (name of participant), this is a song that you like, which reminds you of a place you visited many times,’ then the song would start playing.

The system was calibrated to start playing participants’ music using their live HR data and a machine learning model constructed in a separate project using HR data from a cohort of healthy participants (*n* = 15 > 60 years of age) who underwent the Manheim Multicomponent Stress Test (MMST) (Di Campli San Vito et al., 2023).

The study was funded by the Engineering and Physical Sciences Research Council and granted regional ethical approval by the London – Queen Square Research Ethics Committee. All participants had the capacity to give written, informed consent to participate, as did all caregivers who participated.

## Findings

### Recruitment, retention, attrition

**Figure 3:**
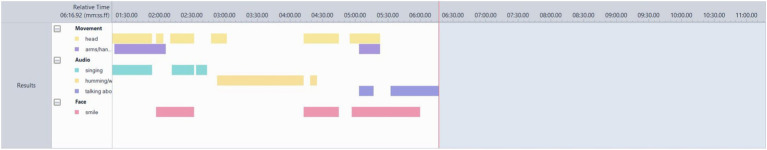
Participant 2 video analysis responses to the song ‘Happy Together’ by The Turtles.

See Figure 3 for a summary of recruitment and retention and Table 1 for gender and age distribution and MMSE descriptive statistics.

**Table 1:** Participant gender and age distribution, mean MMSE.

Gender	Male (%)/Female (%)	12(48)/13 (52)
Age	Mean age (SD: min., max.)	73.8 (7.9: 59. 94)
MMSE	MMSE mean (SD: min., max.)	24 (6.3: 11. 30)

A total of 25 people living with dementia completed a top five playlist. Twenty-one participants completed all sessions offered. Participant 22 was withdrawn due to rapid disease progression and an increase in severity and frequency of agitation during and between sessions.

Reasons that people gave for not joining the study included other illness in the family, spouse felt symptoms were too mild or too severe, too busy, no interest in music and/or radio, unable to encourage spouse (person living with dementia) to join any research, not interested in joining as they were given the wrong diagnosis and did not have dementia, denial by the person living with dementia, which was communicated by them or their spouse in a telephone conversation to the researcher, that they had the disease, and MMSE score falling below the eligibility criteria. Some who were invited gave no reason for declining the study.

### Stage 1 aim 1 findings

#### PCPI co-designed music compilation interview form

Participants were all able to respond to questions formulated for the compilation form and procedures put forward by the PCPI group for this purpose. Not all questions in the form were needed for each participant, as there was some variation in each person’s recall of personally meaningful music. Some participants quickly named music and artists, while others, once an initial piece of music was played, were able to make further selections by listening to related music in terms of genre, artist, and period of commercial release, or by answering questions in the form.

### Stage 1 aim 2 findings

#### Daily agitation data from participants to improve the music activation

The daily agitation logs and the methods to gather the data were not successful. This resulted in no refinement of the algorithm, which was intended to calibrate the system to interrupt the radio with participants’ playlists when HR data indicated stress or agitation, a test we aimed to conduct in Stage 2 (see below). Therefore, playlists were being activated based on the previous algorithm created with data from the healthy cohort (Di Campli San Vito et al., 2023).

Three participants attempted to follow the protocol and reported that they either forgot to complete the logs/or did not remember to press the ‘agitation start’ button on the laptop. They also explained that they did not feel that the term ‘agitation’ was a good fit for the symptoms that they experienced (see discussion). When asked by the researchers how they would describe them, one responded, ‘I feel like I’m shrinking,’ and another described, ‘I feel like my throat is tightening.’ Whilst a caregiver observed how her spouse would become quiet, distant, and less active, as if withdrawing from the world.

### Stage 1 aim 3 findings

#### Participants’ resulting music selections and music listening technology

The top five songs for each participant are shown in Table 2. The playlist selections span classical, pop, rock, jazz, TV, film, theatre, and indie genres across approximately 300 years of music and poetry, with Bach’s Toccata and Fugue being composed in 1703, the hymn ‘Soul of My Saviour’ (participant 8) in 1823, and ‘Jai-Ho’ by the Pussycat Dolls released in 2008.

**Table 2:** Participants’ top five tracks from their playlists (*, did not receive all sessions offered, for example, because symptoms escalated, or participant or spouse illness prevented them from taking place).

Participant/gender/age	Track 1	Track 2	Track 3	Track 4	Track 5	Typical responses
1/F/88	Lady in Red (Chris De Burgh)	Take the A train (Duke Ellington, played by Ted Heath)	Always on my mind (Sung by Shirley Bassey)	Green, green grass of home (Tom Jones)	Strangers on the Shore (Acker Bilk)	Singing along, smiling, laughing, swaying/moving whilst seated
2/M/79	Happy Together (The Turtles)	I’m Still Standing (Elton John)	Jai-Ho (Pussycat Dolls)	Hollow Talk (from TV show ‘The Bridge’)	The Eve of War (War of the Worlds)	Not much movement, smiling
3/M/71	Oh Boy (Buddy Holly)	Where Do You Go To My Lovely (Peter Starstadt)	Singing the Blues (Tommy Steele)	Last Thing on My Mind (Tom Paxton)	All Around My Hat (Steeleye Span)	Singing along, smiling, facial expression showing expressiveness, tapping
4/F/70	Bachelor Boy (Cliff Richard)	Danny Boy (Mario Lanzo)	Will You Still Love Me (The Shirelles)	Unchained Melody (The Righteous Brothers)	Galway Boy (Daniel O’Donnell)	Gentle swaying (seated), occasional smile, some singing
5/F/66	We Are Family (Sister Sledge)	I’m Coming Out (Diana Ross)	Just My Imagination (The Temptations)	Take Another Little Piece of My Heart (Dusty Springfield)	All Around the World (Lisa Stansfield)	Occasional singing, foot tapping, swaying, legs and arms crossed
6/M/70	Walk On By (Dionne Warwick)	What’s Going On (Marvin Gaye)	The Perfect Year (Dina Carroll)	My Oh My (Slade)	Two Can Have a Party (Marvin Gaye/Tammi Terrell)	Foot tapping, finger tapping, smiling, verbal reflections of the song/artist
7/F/71	Bad Habits (Ed Sheeran)	Sound of Silence (Disturbed)	Human (Rag ‘n’ Bone Man)	The Night (Frankie Valli and the Four Seasons)	Lose Yourself (Eminem)	Singing along, moving whilst seated, up and dancing, crying
*8/F/72	Soul Of My Saviour (William Joseph Maher)	Spring (Poem by Gerard Manley Hopkins)	Pied Beauty (Gerard Manley Hopkins)	When Forty Winters Shall Besiege Your Brow (William Shakespeare; Sonnet No. 2)	The Sea and the Skylark (Gerard Manley Hopkins)	Looking down whilst listening, mouthing the words, singing along, swaying
9/F/84	Too Much History (Jack Savoretti)	Impossible Dream (Jack Jones)	Light My Fire (Jose Feliciano)	Stars (Simply Red)	Baker Street (Gerry Rafferty)	Singing, smiling, swaying
10/F/66	Maxwell’s Silver Hammer (The Beatles)	American Pie (Don McLean)	Great Gig in the Sky (Pink Floyd)	You’ve Got a Friend (Carole King)	My Sweet Lord (George Harrison)	Singing, smiling, foot tapping, mouthing words, and laughter
*11/M/67	Let There Be Rock (AC/DC)	Wish You Were Here (Pink Floyd)	Every Day (Slade)	A Day in the Life (The Beatles)	Free Bird (Lynyrd Skynyrd)	Occasional foot tapping, looking at the therapist, sometimes smiling
12/M/69	Okie From Muskogee (Merle Haggard)	Gone Fishing (Chris Rea)	I Know One (Charlie Pride)	Sound of Silence (Disturbed)	I Drove All Night (Roy Orbison)	Occasional singing, nodding head, foot and hand tapping, smiles
13/F/77	I Heard It Through the Grapevine (Marvin Gaye)	I Second That Emotion (Smokey Robinson, The Miracles)	Another One Bites the Dust (Queen)	Three Times A Lady (The Commodores)	It Started with A Kiss (Hot Chocolate)	Singing along, swaying, smiling
14/M/71	Have I Told You Lately (Van Morrison)	Sunshine (Gabrielle)	When The Going Gets Tough (Billy Ocean)	Drop The Pilot (Joan Armatrading)	We Baba Omncane (Busi Mhlongo)	Finger and hand tapping on the table surface
15/M/59	India (Psychedelic Furs)	Rain On Tin (Sonic Youth)	St Stephen (Grateful Dead)	Drill (Wire)	Forget the Swan (Dinosaur Jr.)	Completely still, eyes closed
16/M/86	Look to the Day (John Rutter)	A Mid-Summer Night’s Dream (Medelssohn)	Slavonic Dances: op. 46. No. 2 in E minor (Dvorak)	Eye Level (The Simon Park Orchestra and De Wolfe Music)	Pata Pata (Miriam Makebe)	Head nodding, singing, smiling, conducting with arms and hands
17/F/74	If Tomorrow Never Comes (Garth Brooks)	We Are the Champions (Queen)	7 Years (Lukas Graham)	Short People (Randy Newman)	All You Need is Love (The Beatles)	Smiling, verbal reflections on song meaning
18/M/78	Symphony No. 5 in C Minor (Beethoven)	Requiem, K. 626. In D Minor. Lacrimosa (Mozart)	Toad (Live version: Cream)	Toccata and Fugue in D Minor (J. S. Bach)	Love and Affection (Joan Armatrading)	Occasional foot and hand tapping, singing. Talking over the music
19/F/67	Galway Girl (Ed Sheeran)	If (Bread)	Roll To Me (Del Amitri)	Sweet Baby James (James Taylor)	At Seventeen (Janis Ian)	Frequent arm, leg, and whole-body movements to music whilst seated, and singing
20/M/94	Symphony No. 3 In E Flat, Op.55 - “Eroica” - 1. Allegro (Beethoven)	The Floral Dance (Peter Dawson)	I Could Have Danced All Night (from “My Fair Lady”) (Julie Andrews, Philippa Bevans)	One Day at A Time (Cristy Lane)	Mamma Mia (Abba)	Smiling, foot tapping, finger tapping, verbal reflections on song meaning/memories, talking with partner/family
21/F/67	Perfect Moment (Martine McCutcheon)	Lady in Red (Chris de Burgh)	Isn’t She Lovely (Stevie Wonder)	Hero (Mariah Carey)	When Will I See You Again (The Three Degrees)	Sitting, no movement or singing
*22/F/72	Dancing Queen (Abba)	Fur Elise (Beethoven)	Claire de Lune (Debussy)	Eye Level (Simon Park Orchestra – Them tune for Van de Valk)	Staying Alive (The Bee Gees)	Wandering, occasionally sitting for a few seconds, speaking to husband, stressed
*23/F/73	Seven Little Girls (Paul Evans)	Living Doll (Cliff Richard)	Seamann (Lolita)	These Boots Are Made For Walking (Nancy Sinatra)	All Shook Up (Elvis Presley)	Smiling, singing along, some gentle movement to the music
24/M/69	Popcorn (Hot Butter)	Skymning (Mycket Mera Scout)	Lovely Rita (The Beatles)	Bridge Over Troubled Water (Simon and Garfunkel)	Prelude and the Sound of Music (Richard Rogers)	No singing or movement to the music. Occasional smiles
25/M/77	Are You Lonesome Tonight (Laughing Version) (Elvis Presley)	Dance The Night Away (The Mavericks)	After All These Years (Foster and Allen)	Sacrifice (Elton John)	Non, je ne regrette rien (Edith Piaf)	Smiling, laughter, foot tapping, verbal reflections on song meaning/memories

Some music from other cultures includes ‘Pata Pata’ by Miriam Makeba, ‘We Baba Omncane’ by Busi Mhlongo from South Africa, ‘Skymning’ by Mycket Mera Scout from Sweden, and ‘Seamann’ by Lolita from Germany.

The same composers and pieces chosen by more than one participant included Beethoven (participants 18, 20, and 22), Disturbed performing Simon and Garfunkel’s song ‘Sound of Silence’ (participants 11 and 12), The Beatles (participants 10, 11, and 24), and ‘Lady in Red’ by Chris de Burgh (participants 1 and 21).

The music listening technology summarised in Table 3 shows that radios were present and used in less than half of participants’ homes, with CD players being the most popular (14/25, 56%). More recent technologies are also in use, including music streaming, YouTube (https://www.youtube.com/), and smart speaker technology, which is used by 28% of participants.

**Table 3:** Participant listening equipment grouped by type.

Radio	11
CDs	14
LPs/records/vinyl	3
Music streaming/YouTube	3
Alexa/Sonos/Google	4

#### Examples of participant responses in the video analysis and HR data

We provide some video and HR snapshots of participant responses to specific songs to illustrate how this informed the researchers on whether music selections might have a favourable effect on managing NPS in stage 3 testing. As can be seen, observable responses were not always clear and required supporting information in the form of tagging with feedback on associations and examining HR patterns.

HR data were not collected from the first four participants because the research team had not yet established the best way to do so whilst the first prototype system and algorithm were being built.

Responses from participants were not observed that could be linked to the researchers’ singing along to songs or moving to the music. They did not collect any data on their own movements and look for correlations with participants’ behaviours, as the objective was to determine music that participants would listen to without others necessarily present and whilst listening with them.

#### Participant 2

**Figure 4:**
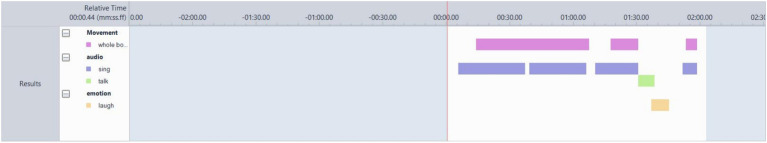
Video analysis of participant 4’s responses to the song ‘Bachelor Boy’ by Cliff Richard.

This was a memorable song for the participant and their spouse from the time they started dating (Figure 3). PT 02 was moving throughout almost the entire song, singing or humming along and smiling. The song brought back memories of when he and his wife first met. Both the discourse from the participant whenever this song was played and the behaviour visible in Figure 4 helped confirm the suitability of this track.

#### Participant 4

Participant four’s husband helped in suggesting songs and music that were meaningful to her, and it was clear when his suggestions were accurate. The researchers recognised the importance of seeking the voice of people living with dementia in confirming choices based on the PCPI data and their personal experiences (Fernie et al., 2024). In Figure 4, she sings and moves to the music – she was not easily able to recall song or artist names, nor to speak, so the singing is a good indication of her arousal increasing and the stimulation of language networks. The movement observed was a gentle swaying to the music. In each session, the participant could become easily distracted, sometimes slightly anxious, for example, thinking that they had to leave the house to pick up their grandchildren, which they never had to do and was something from the past that they used to do. During this song, she was distracted by her husband, who gestured toward the camera at her when she turned to look at him. He playfully did this. She did not understand what he was gesturing, and he said, ‘The camera, you are on camera.’ She then looked at it, laughed and then joined in singing along again. The researcher was not singing, nor was her husband, so this was something she seemed comfortable doing and enjoyed – based on her swaying to the music and her open facial expressions (neither smiling nor frowning, but rather a relaxed expression). These observations suggested that the song was suitable for testing with the RadioMe system, but this did not occur due to delays in preparing the system for testing with these participants.

#### Participant 7

**Figure 5:**
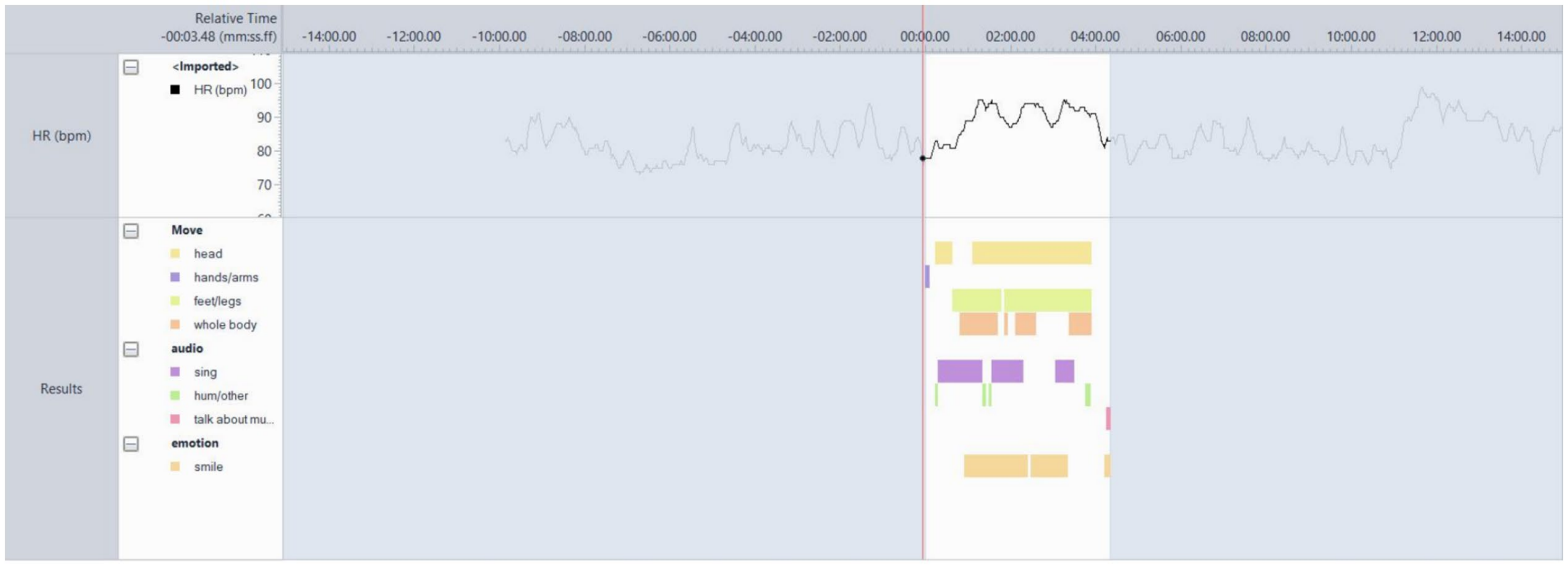
Participant 7’s behavioural and HR responses to ‘Bad Habits’ by Ed Sheeran, typically showing lots of movement, dancing, and HR variability.

Participant 7 often danced energetically to music, which likely caused increased HR activity, but had a complex relationship with some songs and could be moved to tears on occasion due to her associated memories with them or the lyrical content. The song “Bad Habits” (played in several sessions) consistently resulted in behaviours visible in Figure 5, which was taken from Session 10, where it was the first song played after the resting state. The appropriateness of the participant’s music for potentially helping to regulate NPS was indicated mainly by her behavioural responses. This participant did not test the system, as she lived alone for the majority of the time, and it was too complex for her.

#### Participant 11

**Figure 6:**
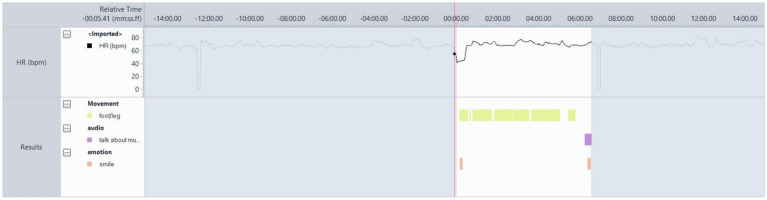
Responses of participant 11 to the AC/DC song ‘Let There Be Rock’.

Participant 11 was seated throughout the session, occasionally crossing and uncrossing his legs. As can be seen in the Observer data (Figure 6), he tapped his foot or feet throughout the song. At the end, he stated, “That’s probably my most favourite song of all time.” The HR pattern exhibits a steep increase at the beginning of the track, with several peaks throughout. Pauses in foot tapping correlate with changes in the music, for example, where the beat is reduced in volume. Due to a lack of observable responses during listening across all sessions, the participant’s comments about the song and the HR pattern helped confirm this as a potentially engaging one to help manage NPS (the participant was uncontactable by the time the system was ready for testing). The participant rarely made clear statements about music that indicated its importance to or impact on him. Sometimes he would talk about early memories of hearing a song and about the artist. Song selection was not straightforward with this participant, as there were no visible behavioural responses. The researcher relied on dialogue that revealed personal preferences and autobiographical associations.

#### Participant 14

**Figure 7:**
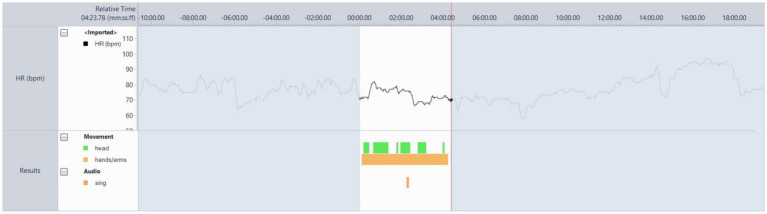
PT-14 Favourite song. After resting, state ‘Have I Told You Lately, by Van Morrison.

Participant 14’s head movement pattern visible in Figure 7 was quite subtle, whilst the hands tapped precisely and audibly on the tabletop, which was typical in all sessions. HR is variable over this song and others, as can be seen, indicating enjoyment throughout the process of selecting and listening, as well as talking in between. The participant did not speak a great deal, which was both characteristic of his personality and symptomatic of his dementia. Most dialogue was about why the music was meaningful and why he liked it. This particular song reminded him of the journeys he took in the car while working on his career. He attended every session with his wife, who helped throughout the weeks by prompting music that she knew he liked. The main indicator of music suitability was what the participant said about the music. They went on to test the system, with feedback all coming from the spouse.

#### Participant 15

**Figure 8:**
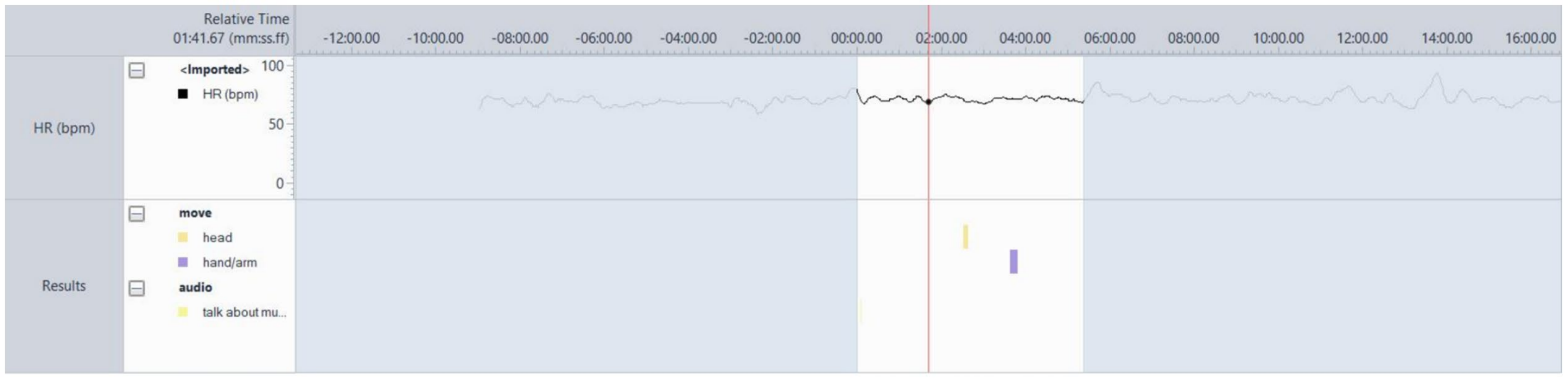
PT-15 Favourite song. After resting, state ‘Forget the Swan’ by Dinosaur Jr. showing hardly any movement, but some variability in BPM values.

PT-15 was typically motionless in sessions (Figure 8), although his legs and feet were not visible due to being seated at a table. In the analysed session (session 10), the researcher (AS) wrote in his notes that he was tapping his foot during the first song after the resting state, ‘Forget the Swan’ by Dinosaur Jr. This was identified as a favourite song. The heart rate is at 80 bpm as the song begins, but drops quickly to 63 bpm, then remains between 75 and 67 bpm for the remainder. Variability in the HR and verbal feedback were the most reliable indicators of music preference. They went on to test the system.

#### Participant 24

**Figure 9:**
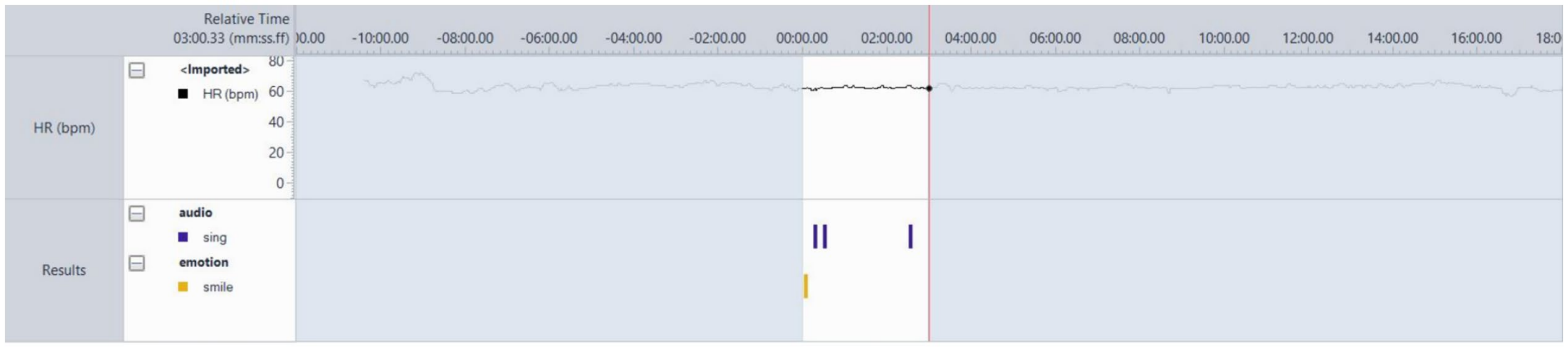
PT-24 S09 preferred song from childhood, ‘Skymring’.

Figure 9 shows the HR activity and behavioural responses that were typical across all nine sessions for participant 24. The only difference is that for this song, which he heard played live and sang along with when camping with the scouts as a boy, he starts to sing along with some of the words. He commented that he remembered the words to the first verse but was less familiar with the other verses. Changes in HR were more evident when we discussed music and research rather than when listening to music. Figure 9 shows some singing to this music from participant 24; however, typically, for them, no tapping or movement to the music, nor any emotional response in their facial expression, was observed. The HR data above the video analysis reflects that there was little movement (acceleration or deceleration). This was the song that elicited the most observable response from the participant, with the singing, and it held the most significant autobiographical associations, which he conveyed in several sessions. This participant completed some testing of the system and provided feedback (Table 4).

**Table 4:** Participant demographics, MMSE screening score at recruitment, and music equipment in their home.

Participant number	Age	Gender	Living with	Diagnosis	Ethnicity	MMSE out of 30	Music equipment/technology used	Attrition
1	88	female	Husband	Mixed Alzheimer’s/vascular dementia	White British	24	Radio, Sunday 12 p.m. show, Radio Cambs. Google Home Hub (for TV), CDs, stereo	N/A
2	79	Male	Wife	Alzheimer’s	White British	28	Alexa, the radio is frequently playing in the kitchen	N/A
3	71	Male	Wife	Other dementias (not known), Primary progressive aphasia (semantic variant)	White British	28	Uses iPad, radio on in kitchen (more used by wife)	N/A
4	70	Female	Husband	Alzheimer’s	White British	14	Alexa (Amazon account)	N/A
5	66	Female	Husband	Alzheimer’s	White British	17	Amazon Echo, the Radio often on in the kitchen and can be heard throughout the house.	N/A
6	70	Male	Alone	Alzheimer’s, Lewy Body, Vascular	White British	11	HiFi/CDs, TV, Freeview Radio	N/A
7	71	Female	Alone	Alzheimer’s	White British	19	CDs	N/A
8	72	Female	Husband	Alzheimer’s	White British	29	Radio, CDs, does not like technology, looking at screens, or background music (radio or other)	Completed enough sessions before leaving for medical reasons
9	84	Female	Husband	Mixed dementia (Alzheimer’s & vascular)	White British	22	TV with YouTube, tablet with Spotify	N/A
10	66	Female	Husband	Alzheimer’s	White British	11	Streaming Service – Deezer CD Player/DVD	N/A
11	67	Male	Wife	Alzheimer’s	White British	28	Watches the same music channel each night on TV. No music in the day. Lots of records, rarely played. Has a Spotify account, rarely used. Has a playlist on phone – rarely used	Uncontactable after 10 sessions __
12	69	Male	Wife	Alzheimer’s	White British	29	Stereo system in conservatory – CDs, radio (one)	N/A
13	77	Female	Husband	Alzheimer’s	White British	13	No music playback system identified	N/A
14	71	Male	Wife	Alzheimer’s	Black (Zimbabwe)	30	CDs, occasional radio	N/A
15	59	Male	Wife	Mild cognitive impairment (MCI)	White British	29	Vinyl, CDs, Cassettes, YouTube	N/A
16	86	Male	Wife	Alzheimer’s	White British	29		N/A
17	74	Female	Husband	Alzheimer’s	White (South African)	30	Uses a phone to listen to music	N/A
18	78	Male	Wife	Alzheimer’s	White British	26	CDs	N/A
19	67	Female	Alone	Alzheimer’s	White British	28	Amazon Echo	N/A
20	94	Male	Wife	Alzheimer’s	White British	28	The participant and his wife listen to CDs daily, and utilise an iPod.	N/A
21	67	Female	Husband	Alzheimer’s	White British	28	Listens to the radio. Has CDs, some of which are in a time capsule she has created for her Alzheimer’s	N/A
22	72	Female	Husband	Alzheimer’s	White British	20	Listens to the radio one day per week	Withdrawn due to rapidly escalating symptoms
23	73	Female	Husband	Alzheimer’s	White British	22	Record player (broken) and singles in the loft – all bought by German dad. Stereo in the garden room. Alexa.	Dropped out due to the husband being unwell
24	69	Male	Wife	Alzheimer’s	White Swedish	30	No listening apparatus	N/A
25	77	Male	Wife	Alzheimer’s	White British	28	CDs, some in a time capsule (box), Amazon Echo	N/A

### Stage 2 findings

#### Activation of music playlists and listening experience

The system algorithm did not enable calibration of the system to play music when participants felt stressed, instead activating playlists at random points. The fidelity of sound was considered better when the external speaker was connected via Bluetooth. The music was enjoyed at times by the participants, but due to its frequent repetition, it became monotonous. Some participants commented on the need for more precise calibration and for longer playlists. Two participants commented on specific music from their playlist having a calming effect. One participant said that they would prefer the system to play the whole track when it was triggered, rather than fading back the radio when HR was reduced. A point was raised about the volume of each track in their playlist being different, suggesting that the system might need to automatically adjust the volume of some music because it was recorded or performed quietly. For example, ‘Galway Girl’ is perhaps louder due to being a livelier song, whereas ‘Only A Woman’s Heart’ has a gentler tempo and drum track, with soft strings. One participant suggested that the music’s profile should match the listener’s HR at the time of listening.

The spouse of a participant felt that spoken introductions to the music (pre-recorded into the system by the researchers prior to installation) would help create a pause and gain attention.

Some preferred not to have the radio on, or for it to be playing less, or for more choice of radio stations. Others felt that the playlists interrupted their enjoyment of the radio.

#### System operation

The average installation period was 5.1 weeks, with the shortest being one week. Participants were able to connect the watch to the laptop for extended periods, but this often took several minutes and was unstable. The Quickstart guide was sufficient to start the radio playing, and playlists were already assigned to each participant, so this did not need to be selected. One participant requested that the system be uninstalled, as it was too complex to operate and unreliable. Feedback indicates that some found the watch too awkward to operate due to the small icons and the fiddly swipe and button presses required to activate the RadioMe app. Despite the various operational struggles experienced by both participants and researchers, none of the equipment, whether hardware or software, was damaged.

## Discussion

### Summary

We have reported on the recruitment, data collection, music compilation process, and behavioural responses of participants whilst listening to their musical selections in their homes with researchers present. The music was compiled with the intention of using it at a later stage of development to understand its effects on agitation. Some preliminary findings have been presented on one component of the RadioMe system (Stage 2), where playlists interrupt radio broadcasts based on changes in live HR from smartwatches. The calibration for this was based on an algorithm developed in a separate project using data from a healthy cohort whilst they underwent a standardised stress test.

### Recruitment, playback equipment, and the compilation process

The initially required target sample of participants was higher, but due to the global COVID-19 pandemic interruption, this number was reduced. The Join Dementia Research platform was an efficient method for identifying eligible participants. Black, Asian, and ethnic minority representation was absent, with predominantly white British or European participants (Table 4). The geographical region over which recruitment took place is populated by diverse cultures; however, these cultures were not visible on the Join Dementia Research platform, which covered the area. This has been cited as a common occurrence, and solutions are being developed to increase inclusion (Brijnath et al., 2022).

Music equipment was quick and easy to set up for the researchers, with occasional disruptions in music streaming due to poor internet connections, which in one or two cases led the researchers to download music prior to sessions, as the participant had expressed a preference for it during their previous visit.

The researchers’ observations indicated that participants enjoyed listening to and discussing their music and the memories associated with it, a finding supported in the literature (Gerdner, 2000; Lineweaver et al., 2022).

They were engaged in the process, i.e., thinking about why the music was important to them based on their relationship with it and their choices and on the aesthetic appeal to them of the music, the artist performing it, and the arrangement.

It cannot be confirmed how relevant different versions of a song might be for the purposes of managing NPS, but the researchers found that in some cases, different versions were discussed and selected. Some research has reported that preferred and non-preferred music are equally beneficial in helping to manage agitation (Zare et al., 2010), but has not investigated preference for specific versions. Researcher-selected music has been described as equally effective in reducing challenging behaviour in dementia when compared to playlists compiled through in-person interviews (Kulibert et al., 2019). Whilst personally meaningful playlists have been effective in managing anxiety and relationship strain in dementia (Julian Maria Paisley, 2014) in relation to comparators or usual care, as well as managing other symptoms (Thomas et al., 2017), there appear to be instances where neuropsychiatric symptoms can be influenced by less specific playlists.

According to some research, older listeners have the broadest tastes due to their exposure to music across their lifespan from various genres and niches (Krumhansl, 2017). However, the researchers were surprised by the diverse range of music selected by individual participants, the wide variety of genres across all of them, and how some of the music had gained significance for them at various points in their lives over many decades.

Although beyond the scope of discussion in this article, if a system like RadioMe were to be fully developed, it would ultimately need to support the music preferences of people from all nationalities and cultures. Whether any limitations are presented when using the streaming platforms reported here for finding such music remains to be seen. Finding the track ‘Skymning’ was not straightforward, and there were other instances where music videos could be found on YouTube, but not the audio recordings via other streaming services.

### MMSE scores and their interaction with participants’ ability to identify and listen to music

The threshold of ≥10 in the MMSE score (all are shown in Table 4) seemed appropriate for inclusion, with participants scoring 11–14 still able to identify music and discuss their association without the need for support. Exceptions where inclusion criteria might warrant re-examination are those where symptoms rapidly progress, as is the case in certain types of dementia, including Creutzfeldt–Jakob disease. Literature is available on their diagnosis and management, which may be helpful for future engineering projects of this nature (Hermann and Zerr, 2022).

### Daily agitation logs, participants’ experience of agitation, and biodata collection methods

The progression from an algorithm built with HR data from the healthy cohort to one using participant data relied on the daily agitation logs, and this method was not feasible. Some comments relayed by participants to the researchers reported that in a moment of stress or anxiety, for example during a difficult conversation/discussion between spouse and caregiver, it was not possible for either of them to stop or remember to stop the discourse or flow of events so that they could note down a description of their feelings and behaviours and all other required details whilst simultaneously pressing a button on the laptop.

A further confounding factor was participants’ experience of symptoms, which did not necessarily equate to agitation. This may have implications for how a system like RadioMe might function, potentially requiring more refined calibration with HR data gathered from people living with dementia experiencing more subtle forms of stress and disorientation. Agitation, as well as other NPS, including depression, anxiety, irritability, and sleep impairment (Lyketsos et al., 2002), might manifest differently in terms of severity and frequency at the earlier stages of dementia, when people are still living at home. Agitation is a symptom described as one of several termed ‘behaviours that challenge’ and includes physical and verbal aggression (James et al., 2023). It has also been defined as behaviours that communicate unmet physiological or psychological needs (Stokes, 2005). Whilst agitated behaviours clearly merit focused research to help with their management, looking at them in isolation and measuring treatment-related effects can be problematic, since the causes have been linked to other symptoms, including delirium, depression and cognitive decline (James et al., 2023), which also need addressing.

A relevant point in the discussion of potentially using music in the home to help manage NPS is how to identify milder symptoms so that music can be played at the appropriate time and become assimilated into people’s lives in advance of more serious and frequent symptoms developing. The Cohen-Mansfield Agitation Inventory (Cohen-Mansfield et al., 1989), which is used in many agitation-focused studies to screen participants and measure treatment-related effects, was designed to detect more severe symptoms. More sensitive screening questions might be required to detect earlier, more subtle ones (Bateman et al., 2020) that are experienced by people still living at home and that require interventions to help manage them. Some studies have used behaviour mapping (Cohen-Mansfield and Werner, 1997; McCreedy et al., 2019), which may serve as an alternative to the CMAI, operating in parallel with biodata collection (Kleine Deters et al., 2024). The Agitated Behaviour Scale may also be a more sensitive and reliable option for identifying the threshold at which music should be used or activated (Alberta Health Services, n.d.).

Heart Rate Variability (HRV) might also provide a method for detecting agitation. Some studies have reported HRV decreases in dementia, which could be indicative of stress (Wang et al., 2025), but further research is required to determine the reliability of such methods.

For reliable biodata collection suitable for detecting the onset of behaviours such as agitation, some human involvement in observations is necessary (Kleine Deters et al., 2024). This has been tested, revealing it as a potentially reliable method (Nesbitt et al., 2018). Other literature supports the need for HR to be recorded during moments of anxiety, frustration and relaxation, together with behavioural observations (Khan et al., 2018), which may present challenges in the home setting, as was the case with the RadioMe daily agitation logs.

A systematic review of technology for detecting NPS in dementia reports that there is great potential for sensor technology (Husebo et al., 2020), which would effectively gather behavioural data. Some of the research included robots that assess social and emotional responses, as well as a text analysis tool for detecting changes in patterns of communication between people living with dementia and others. Perhaps most relevant to the objective of RadioMe is the use of sensor rays situated in living environments that detect behavioural and routine changes (Hattink et al., 2016). The system could notify caregivers of significant changes in behaviours – perhaps such sensor data could also be used in algorithms to activate music playlists and help prevent escalating stress or agitation.

### Session data analysis and observations

The video analysis helped identify the range of responses when a person listened to music, from no detectable changes in body or face to subtle expressions, laughter, crying, and vigorous movement or dancing. Whilst video analysis has been employed in dementia and music research, for example, helping to identify its influence on communication and interactions (Clare et al., 2020), the researchers did not detect any advantages specifically for the playlist compilation process described here that would justify the time required to conduct it.

The HR behaviours observed in sessions and during video analysis between sessions did not reliably inform on music responses. The researchers discussed them in the context of the literature, and, where observable behavioural responses were absent, they looked to the HR activity and considered whether it might indicate enjoyment of the music. HRV can be significantly impacted by music (Ellis and Thayer, 2010) and serves as a good marker of both emotional state (Fourie et al., 2011) and mental state (Howells et al., 2014).

Whilst the visible HR patterns may have been indicative of increased HRV during listening, there was no formal analysis of these data, and the listening conditions were not designed to answer HR/HRV-related research questions. There is still insufficient evidence to establish whether, for example, increased HRV equates to reduced stress and increased relaxation whilst listening to music in any population, including those with dementia, and further research is warranted in this field (Koelsch and Jäncke, 2015).

### Outliers

Participant 8 chose to attend all sessions and consistently expressed a dislike for pre-recorded and background music, talking almost exclusively about poetry and environmental sounds such as birdsong. The researcher used the assessment of music preference form and processed it each week, but the participant would not engage for long and consistently made her preferences and dislikes clear.

As with any participant who could initially be considered as an outlier, their inclusion is important (Phoenix and Orr, 2017), and this person contributed to more thoroughly determining whether there was any music or recorded poetry that, if tested as part of the RadioMe system, might induce a sense of calm, relaxation or excitement. Inclusion might help identify other modifications to the music compilation process or system design, potentially increasing user accessibility and utility.

The participant was animated and engaged in every session, but predominantly when we read poetry. She reflected on its meaning and content, as well as her associated memories. When the music therapist played classical guitar music, she appeared relaxed yet perplexed in her facial expression and posture, which may have confirmed a preference she had expressed on several occasions for live-performed acoustic music.

### Potential alternatives to music listening for NPS management

People who favour poetry or literature over music may benefit from its use/delivery in terms of mood, arousal, and psychological wellbeing. There is some research comparing audiobooks with music in stroke (Sarkamo et al., 2008) and reading for people living with dementia (Baker et al., 2021; Guétin et al., 2009), which may help inform further development alternatives. Further study findings indicate that conversations recorded by family members for their relatives with dementia can also be effective in reducing physically and verbally aggressive behaviours (Garland et al., 2007).

### Early-stage testing and HR-driven music activation

The RadioMe system testing in Stage 2 clearly indicates that it needs to be easier to operate, which corresponds with other findings and recommendations for technology development for older people (Heart and Kalderon, 2013; Peeters et al., 2016). There are further implications for people living alone with dementia, as illustrated in participant 7, who could not operate the system alone, and there were no others in the household who could have helped. The music playlists should not become repetitive, and the activation of the music needs to be better calibrated, which may be achieved by using HR data from people living with dementia rather than healthy cohorts. Other options for data collection have been suggested under ‘Potential Alternative for Collecting Biodata Behavioural Data for Calibration of Music Activation’, such as motion sensors.

### Limitations

It is clear that at this stage of development, RadioMe is not yet ready for testing to determine whether it can deliver the right music at the right time and for the necessary duration to achieve NPS management. If biodata from wearable devices is to be used to regulate music delivery, a range of reliable sensors needs to be combined, and signatures for stress, anxiety, agitation and all NPS need to be clearly identified (Khan et al., 2018). Other sources have reported that more research is needed to confirm the correlations between HRV and HR and agitation in dementia (Liu et al., 2015), including studies of autonomic function prior to, during, and following episodes of agitation (Ostergaard, 2025). This should be integrated into an easy-to-operate system and tested in real-world, home-based situations to identify anomalies and account for outlier data (Kleine Deters et al., 2024).

The daily agitation and manual pressing of the agitation button on the laptop where RadioMe was housed did not work. This was intended to provide HR data to refine the music activation algorithm. Modifications to the protocol might have enabled the collection of useful behavioural data to refine the music activation algorithm. For example, a more precise and easier-to-follow protocol for recording HR and behavioural observations could be developed by discussing with participants whether there are regular times when stress levels might be high and gathering data at those points. Although some attempts were made by the researchers to do this, it was conducted as a problem-solving exercise rather than being developed and tested as a protocol. Alternatives suggested earlier in this discussion may yield useful data for the music activation algorithm.

The involvement of the PCPI group in co-designing the music compilation methodology was beneficial, and their input was intended to advise on user interface and functionality modifications once the initial prototype and successful testing of the music activation algorithm were completed. Input from the group and participants would have been helpful, even at this stage of development, as has been reported in the literature on the co-design of technology for older people and those living with dementia (Müller-Rakow and Flechtner, 2017). Co-designing with older people and those living with dementia requires a clear outline, including the identification of barriers, facilitators, and roles, to achieve realistic and achievable goals (Sumner et al., 2021).

The music compilation process could be completed with fewer sessions and without the use of video and HR whilst listening, which would speed up the process. Although some of these data, for example, the video from participant 4 and HR from participant 11, were referred to as a guide to selecting music, they were secondary to the personal music choices of participants and their associations with them – the latter being particularly evident for participants 14 and 24 and reported in related literature (Fernie et al., 2024).

A limitation of the playlist testing process – specifically the listening whilst gathering video and HR – is that participants were listening with researchers, and this did not replicate conditions under which listening would take place using the RadioMe system, i.e., they would not be sitting specifically to compile and listen to music, and they would not be accompanied by researchers. They would be going about their daily life. Therefore, to some extent, the researchers were unable to fully establish whether the playlists would have the desired effect during the proposed Stage 3 testing. For participants who were moving whilst listening, particularly participant 7, HR would have increased due to physical activity, skewing any data that could have indicated specific HR or HRV responses purely from listening.

The HR data collected during listening was not properly analysed to determine their effect on arousal, nor was a listening protocol implemented to assess playlists for consistent responses. This limitation reduces the usefulness of the HR data, resulting in poor utility for informing listening effects.

### Current developments and future directions for music listening delivery and NPS management

Person-centred approaches to music selection have been delivered manually at the time of agitation onset; however, this requires someone to be present, observing, and able to recognise the external behaviours indicative of onset that are unique to each person (Lineweaver et al., 2022). What is still not known is whether the methods described and the technology used by people living with dementia and the wider population – including publicly available streaming services with mood functions – could converge in ways suggested in some literature (Schriewer and Bulaj, 2016) and system operation and interfaces designed to overcome accessibility barriers (Vidas et al., 2024).

The benefits of understanding the responses of people with dementia when listening to liked and disliked songs and music include refining algorithmically driven music selection based on genre and potentially avoiding adverse effects associated with disliked music. The refinement could be based on memories and associations being factored into the algorithm, or even live HR data, so that desired arousal states could be reached (Sice et al., 2020). Such additional knowledge could also make group music listening activities safer and more enjoyable for people with dementia, reducing the risk of stress or upset through inadvertent playing of disliked music.

If a functional prototype of the RadioMe system can be achieved, there must be options for users to override the agitation detection algorithm, as indicated in some of the user testing feedback. They may wish to listen to the radio despite the system detecting stress; for instance, they could be listening to a programme such as a political debate, which may agitate them but which they want to engage with. More advanced and sensitive behavioural observation technology, such as that earlier suggested (Hattink et al., 2016; Husebo et al., 2020), may enable systems to distinguish between these behaviours and be used in algorithms to decide whether agitation onset has occurred or heightened arousal and engagement with a desired stimulus.

If RadioMe or a similar system were to become available, users could update their playlists as they prefer—a point raised by one system-testing participant—and this should not require a process as lengthy as that undertaken by researchers here, who delivered 15 sessions. Systems have been developed that gradually induce relaxation (Guerrier et al., 2021), match music to heart rate, or map it onto a valence to achieve desired states of arousal (Chen et al., 2022; Sice et al., 2020). These could be integrated into automated systems to help manage NPS and maintain quality of life and independent living for as long as possible.

## Data Availability

The datasets presented in this article are not readily available because they comprise video data of the participants listening to their music and as such cannot be made available. Questions regarding the datasets should be directed to the corresponding authors.
