# Supplementary material for: RadioMe: an automated home-based radio, music playlist, and diary reminder system: Report on recruitment, music compilation, and listening, and preliminary testing of heart rate activated music
Source: Front Psychol. 2025 Oct 24;16:1627466. doi: 10.3389/fpsyg.2025.1627466 (PMC12592169; doi:10.3389/fpsyg.2025.1627466)
Supplement: Supplementary file 1 [file Supplementary_file_1.docx]

## Appendices

### RadioMe assessment of music preference form questions:

1. Do you have any records or CDs that you particularly enjoy?
2. What music do you enjoy most? *(Vocal, instrumental, may include hymns, folk, popular, opera, symphonic movements, anthems, etc)*
3. Some people find certain music relaxing, is there any that you might find relaxing or calming? *(Prompts: Classical, ambient, hymns, Gregorian chant or other vocal/instrumental)*
4. Some people listen to particular music and it can make them feel a little sad *(is there any that makes you feel sad/upset and would rather not hear?)*
5. Do you like these or any other genres of music:
6. Pop 8. Hymns
7. Rock 9. Blues
8. Heavy metal 10. Funk
9. Dance 11. Classical
10. Electro 12. Folk
11. Rap 13. World (from other cultures)
12. Reggae 14. Northern Soul
13. Jazz

1. Do you have a favourite singer, composer or band?
2. Music sometimes plays an important part in film and television (can you think of any that you enjoy?)

*To help trigger memories of music, consider genre: action, romance, comedy, fantasy, biographical. Might the film/program be memorable due to the story, the actors, setting, location, etc*

1. Are there any other songs/pieces of music from any occasions/events that you like? (Birthdays, Christmas, holidays, significant life events such as getting a job, passing an exam or driving test)
2. Is there any music that reminds you of experiences of travel, work or living abroad or in a particular region?
3. Do/did you (a family member) play a musical instrument? (*Yes-No: which instrument)*
4. Do/did you enjoy singing? (Yes-No, details)
5. Did/do you enjoy dancing? Yes-No/details: (Type, period, venue, with who)


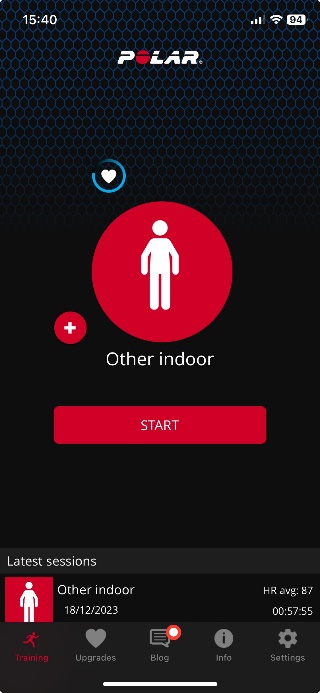


Figure 1. Screenshot showing Polar app and start button, which was in shot of the video to facilitate synchronisation to the video in the Noldus Observer analysis software
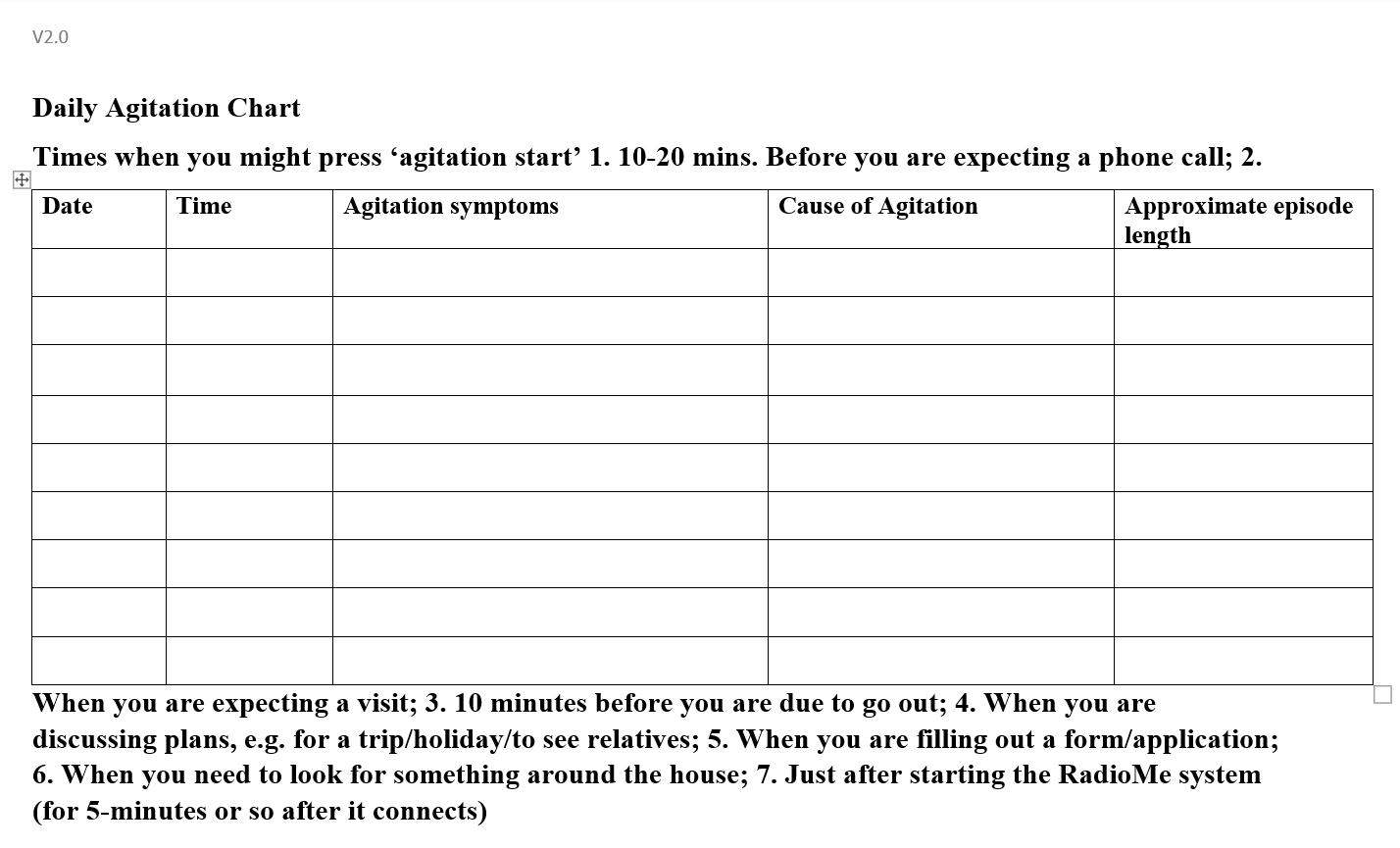
Figure 2. Daily agitation chart


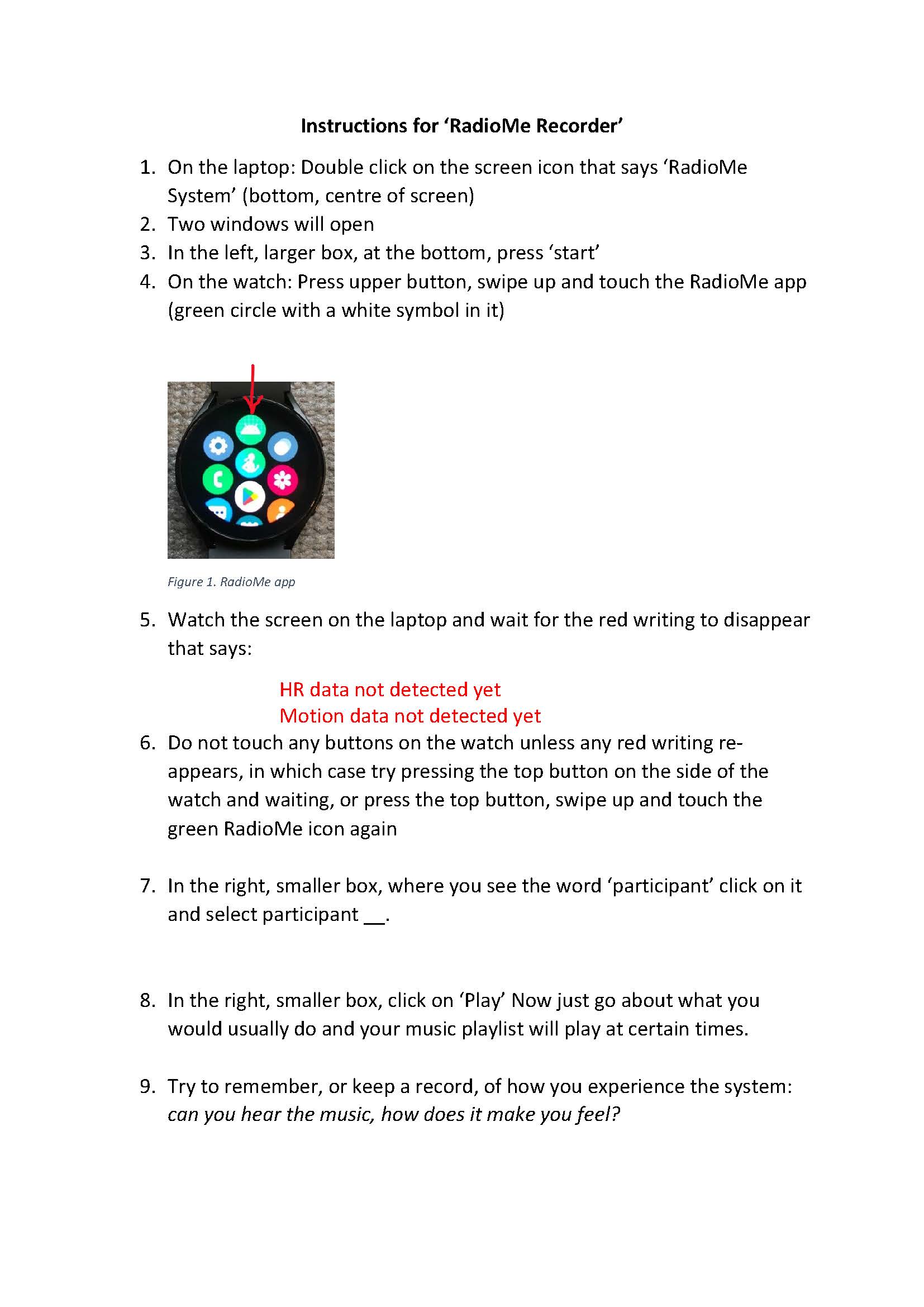


Figure 3. Quickstart guide for the RadioMe system
